# Move in nature, get strong with smart sports: physical activity habits and health-related psychological factors among adults exercising in green and smart sports environments

**DOI:** 10.3389/fpubh.2026.1908291

**Published:** 2026-07-22

**Authors:** Mahmut Ulukan, Nisanur Bingölbalı, Mehmet Tuncer, İzzet Karakulak, Kerim Rüzgar, Okan Sarı, Serdar Koç, Mehmet Ilkım, İsmail Belli, Faik Öz, Levent Ceylan

**Affiliations:** 1 Department of Recreation, Faculty of Sports Sciences, Istanbul Gelisim University, Istanbul, Türkiye; 2 Department of Sports Management, Faculty of Sports Sciences, Fırat University, Elazığ, Türkiye; 3 Ministry of National Education, Ankara, Türkiye; 4 Department of Sports Management, Faculty of Sports Sciences, Mardin Artuklu University, Mardin, Türkiye; 5 Iluh Anatolian High School, Ministry of National Education, Batman, Türkiye; 6 Department of Exercise and Sport Education for the Disabled, Faculty of Sports Sciences, Inonu University, Malatya, Türkiye; 7 Department of Exercise and Sport Sciences, Faculty of Sports Sciences, Haliç University, Istanbul, Türkiye; 8 Faculty of Sports Sciences, Avrasya University, Trabzon, Türkiye; 9 Faculty of Sports Sciences, Hitit University, Çorum, Türkiye

**Keywords:** green exercise, perceived stress, physical activity, psychological resilience, quality of life, smart sports environments, sport motivation, structural equation modeling

## Abstract

**Introduction:**

Sedentary lifestyles threaten physical and psychological well-being. Nature-integrated green sports environments and digitally supported smart sports environments may promote physical activity and sustainable health. This study examined the direct and indirect associations among green facility exposure/use, smart facility/technology interaction, physical activity, sport motivation, perceived stress, psychological resilience, and quality of life.

**Methods:**

A quantitative, cross-sectional correlational design was used with 300 adults living in Türkiye (168 women and 132 men; mean age = 33.4 years, SD = 9.7). Data were collected online between 22 May and 14 November 2025 and analyzed using a hybrid structural equation model combining observed composite indices and latent psychometric constructs.

**Results:**

The model showed acceptable fit (*χ*^2^/d*f* = 2.10, CFI = 0.96, TLI = 0.95, RMSEA = 0.051, SRMR = 0.044). Physical activity was positively associated with sport motivation (*β* = 0.48, *p* < 0.001), which was positively associated with psychological resilience (*β* = 0.42, *p* < 0.001) and quality of life (*β* = 0.27, *p* < 0.001). Green facility exposure/use (*β* = 0.18, *p* < 0.05) and smart facility/technology interaction (*β* = 0.12, *p* < 0.05) were positively associated with physical activity. Perceived stress was negatively associated with psychological resilience (*β* = −0.33, *p* < 0.01) and quality of life (*β* = −0.29, *p* < 0.01). Indirect associations linked physical activity with quality of life through sport motivation and psychological resilience.

**Discussion:**

Green and smart exercise contexts may support physical activity and related motivational and psychological processes. Because the study was cross-sectional, the findings should be interpreted as associations rather than causal effects. Nature-based and technology-supported opportunities may inform sports-environment planning and public-health strategies.

## 1 Introduction

Inactivity associated with modern lifestyles has become one of the most serious public health challenges of the contemporary era. Digitalization, urbanization, and technology-driven sedentary routines have reduced daily movement and negatively affected both physical and mental health (1). A sedentary lifestyle leads to chronic health problems such as obesity, cardiovascular disease, and diabetes, as well as psychological consequences such as stress, anxiety, and low life satisfaction (2). In this context, new solutions are needed to guide individuals toward sustainable health behaviors. Green, nature-integrated, and digitally supported sports facilities have emerged as promising contexts for promoting physical activity and supporting well-being. In Türkiye, the increasing prevalence of large-scale urban green projects and public open spaces that include walking–cycling routes and sport areas makes the “green facility” approach highly relevant in practice; moreover, some sites have begun to incorporate technology-supported features such as smart bicycle systems and other digital exercise support tools, indicating a growing “smart facility” dimension.

The World Health Organization (3) emphasizes that at least 150 min of moderate physical activity per week is a fundamental health behavior for preventing chronic diseases and improving quality of life. Recent studies have shown that regular physical activity has positive effects not only on physical health but also on psychological resilience and stress coping skills (4). A meta-analysis conducted by White et al. (5) revealed that physical activity contributes to mental health through mediators such as self-efficacy, psychological resilience, and social support. Recent evidence has also reported a significant positive relationship between physical activity levels and quality of life among adults, further emphasizing the role of active lifestyles in sustainable health promotion (6). Therefore, physical activity is indispensable for the sustainability of both individual health and societal well-being in modern societies. For the purposes of this study, exercise was defined in accordance with WHO guidelines as moderate-to-vigorous physical activity (MVPA), encompassing activities such as brisk walking, jogging, cycling, and vigorous sport engagement that substantially raise the heart rate and increase breathing rate.

Psychological resilience is defined as an individual’s capacity to cope with and adapt to stressful life events and the challenges of modern life. Research indicates that regular physical activity enhances individuals’ quality of life by increasing this capacity (4). A recent study, particularly among university students, found that physical activity levels are positively correlated with psychological resilience and well-being (7). These findings demonstrate the critical role of physical activity in strengthening individual stress-coping mechanisms and sustaining quality of life.

Environmental factors, as well as motivational sources, influence participation in sports and physical activity. Within the context of self-determination theory, fostering intrinsic motivation increases individuals’ participation in sports, while environmental awareness shapes the quality of preferred sports environments (8). The current literature shows that activities in nature, known as “green exercise,” have important benefits for both mental and physical health (9). A demand analysis conducted by Reuß and Huth (10) found that urban residents seek year-round access to green sports spaces and that connecting with nature strengthens their sports habits. In addition, technology-supported exercise environments may enhance self-monitoring, feedback, and engagement, thereby contributing to exercise participation and sustainability. Recent evidence has further suggested that proximity to functional urban parks may be positively associated with park visitation, physical activity, and well-being, highlighting the importance of accessible green environments in supporting sustainable active living (11). Two complementary environmental-psychological frameworks further ground the present investigation. Attention Restoration Theory (ART) (12) proposes that natural environments facilitate psychological restoration by providing respite from directed attention demands, thereby reducing mental fatigue and stress. Stress Reduction Theory (SRT) (13) similarly argues that exposure to natural settings triggers rapid psychophysiological recovery from stress through evolutionary affiliative responses to natural scenes. Together, ART and SRT provide a theoretical basis for the expectation that exercising in green environments confers additional psychological benefits beyond those attributable to physical activity alone.

While the effects of physical activity on health have long been studied, research on the holistic effects of green and smart sports facilities on individuals’ motivation, stress, psychological resilience, and quality of life remains limited. Furthermore, the existing literature lacks research on integrating nature-based (green) exercise settings with technology-supported exercise features (14). Despite growing interest in both green exercise and technology-supported physical activity, studies examining their joint relationship with physical activity and downstream psychological outcomes remain limited. In particular, the literature lacks integrative models that consider green exercise exposure, interaction with smart technology, motivation, stress, resilience, and quality of life within a single analytical framework (14). The integration of green and smart exercise contexts within a single conceptual framework is motivated by contemporary exercise behavior patterns, where physical activity increasingly occurs at the intersection of natural and digital settings. Examining both contextual dimensions together allows assessment of their relative and combined associations with physical activity and downstream psychological outcomes. The novel contribution of this study lies not merely in replicating known PA–health pathways, but in examining whether green facility exposure and smart technology engagement add contextual variance in physical activity and whether the established motivational and resilience pathways operate similarly within this integrated contextual framework. Smart sports environments are defined as exercise spaces and contexts that integrate digital technologies—including wearable tracking devices (e.g., smartwatches, fitness bands), mobile fitness applications, app-based workout guidance, and data-driven monitoring systems—to support and enhance physical activity behavior and user engagement.

In the present study, the “green” and “smart” components were operationalized through participants’ self-reported use and exposure indicators so that both concepts were directly represented in the analytical model. Green sports facility exposure/use was assessed with a brief set of items capturing how often participants exercise in nature-based environments (e.g., parks, green corridors, outdoor green sport areas) and the perceived relevance of these settings for their routine. Smart facility/technology interaction was assessed with a brief set of items reflecting engagement with technology-supported exercise features, such as wearable/app-based tracking and perceived usefulness of digital exercise support tools (e.g., app-based guidance and, where available, QR-based information boards or smart bike systems). This operational approach enabled examination of whether green and smart exercise contexts are associated with physical activity and related psychological outcomes within a single structural model.

Accordingly, the present study tested a structural model linking green facility exposure/use, smart facility/technology interaction, physical activity, sport motivation, perceived stress, psychological resilience, and quality of life. More specifically, it aimed to examine both the direct relationships among these variables and the indirect path-ways through which green and smart exercise contexts may be associated with quality of life. It was expected that green facility exposure/use and smart facility/technology interaction would be positively associated with physical activity, that physical activity would positively predict sport motivation, that sport motivation would positively predict psychological resilience and quality of life, and that perceived stress would negatively predict psychological resilience and quality of life.

## 2 Materials and methods

### 2.1 Research design

This study employed a quantitative, correlational research design to examine the relationships among green facility exposure/use, smart facility/technology interaction, physical activity level, sport motivation, perceived stress, psychological resilience, and quality of life. Structural Equation Modeling (SEM) was used to test the direct and in-direct associations among the study variables, including the proposed mediation pathways (15, 16).

### 2.2 Participants

The target population consisted of adult individuals living in Türkiye who engage in physical activity and report exposure to green exercise environments and/or technology-supported exercise practices. A convenience sampling method was used be-cause of accessibility and feasibility considerations.

Inclusion criteria were being 18 years of age or older, residing in Türkiye, and voluntarily agreeing to participate by providing informed consent.

The final sample included 300 participants (168 female [56%], 132 male [44%]) aged 18–65 (*M* = 33.4, SD = 9.7). The sample size met SEM requirements and was considered adequate for the estimated structural model. Participants were also required to provide information regarding their exposure to green exercise environments and/or technology-supported exercise practices via the GFE and SFTI indicator items. *A priori* power analysis was conducted using G*Power 3.1. Based on a medium effect size (*f*^2^ = 0.15), *α* = 0.05, and desired power of 1 − *β* = 0.80, the minimum required sample size for the present number of estimated parameters in the SEM was N = 255. The obtained sample (*N* = 300) exceeded this threshold, confirming adequate statistical power for the planned analyses.

### 2.3 Research procedure

This study adhered to ethical standards and academic research protocols. After the research objectives had been defined and the variables operationalized through a literature review, the measurement tools were selected accordingly. Ethical approval was granted by the Istanbul Gelisim University Ethics Committee on April 18, 2025 (Decision No: 2025-06-68). Data collection occurred between May 22 and November 14, 2025, via a structured Google Forms questionnaire. The survey link was disseminated through social media, email, and instant messaging channels. Participants were informed about the study’s purpose, voluntary nature, and confidentiality. Informed consent was obtained digitally prior to survey access. The questionnaire included demographic items and validated scales detailed below.

### 2.4 Measures

#### 2.4.1 International Physical Activity Questionnaire-Short Form (IPAQ-SF)

The International Physical Activity Questionnaire–Short Form (IPAQ-SF) is a widely used standardized self-report instrument for assessing physical activity in population-based research (17). The Turkish version of the IPAQ-SF was adapted and psychometrically validated by Savcı et al. (18) in a large sample of university students aged 18–32. The scale includes 7 items assessing the frequency and duration of walking, moderate physical activity, vigorous physical activity, and sedentary behavior over the past 7 days. Each activity is assigned a MET (Metabolic Equivalent of Task) value (walking = 3.3 METs, moderate = 4.0 METs, vigorous = 8.0 METs), allowing for calculation of total physical activity in MET minutes/week. In the Turkish validation study, the scale demonstrated strong construct validity via factor analysis, and high test–retest reliability, with Pearson correlation coefficients ranging from *r* = 0.62 to 0.91 across forms. Internal consistency was also acceptable (Cronbach’s *α* = 0.82). The scale classifies individuals into three physical activity categories: inactive, minimally active, and highly active based on standard MET thresholds. Cultural adaptation was conducted through forward and backward translation by bilingual experts, followed by cognitive interviews and pilot testing with Turkish-speaking participants. The international IPAQ group approved the instrument and is widely used in both clinical and public health research in Türkiye.

#### 2.4.2 Sport Motivation Scale-II (SMS-II)

Developed by Pelletier et al. (19) and adapted into Turkish by Öcal and Sakallı (20), this 18-item scale assesses different types of motivation based on Self-Determination Theory. It includes six sub-dimensions: intrinsic motivation, integrated regulation, identified regulation, introjected regulation, external regulation, and amotivation. Items are rated on a 7-point Likert scale ranging from 1 (not at all) to 7 (completely). Higher scores indicate higher motivation levels. The Turkish version demonstrated good psychometric properties, with Cronbach’s alpha values ranging from 0.78 to 0.89 across sub-dimensions.

#### 2.4.3 Perceived Stress Scale (PSS-10)

The Perceived Stress Scale (PSS-10), originally developed by Cohen et al. (21), was adapted into Turkish by Eskin et al. (22). This scale measures the degree to which situations in one’s life are appraised as stressful. It contains 10 items (five positively and five negatively worded) rated on a 5-point Likert scale (0 = “Never” to 4 = “Very Often”), with positively worded items reverse-scored. The Turkish version showed high reliability (Cronbach’s alpha = 0.86) and acceptable construct validity, as indicated by confirmatory factor analysis (CFI > 0.90, RMSEA < 0.08).

#### 2.4.4 Brief Resilience Scale (BRS)

The Brief Resilience Scale (BRS), originally developed by Smith et al. (23) was adapted into Turkish by Doğan (24) to assess individuals’ ability to recover from stress and adversity. The adaptation aimed to ensure the scale’s reliability and validity within a Turkish university student population. The scale consists of 6 items, 3 positively and 3 negatively worded, rated on a 5-point Likert scale from “strongly disagree” to “strongly agree.” Items 2, 4, and 6 are reverse-scored. The Turkish adaptation study demonstrated that the scale had good internal consistency (Cronbach’s alpha = 0.83), a clear single-factor structure, and strong construct validity. Positive correlations were observed with life satisfaction, self-efficacy, and hope, while negative correlations were found with depression and negative affect. These findings confirm that the Turkish version of the BRS is a reliable and valid instrument for measuring psychological resilience, particularly among university students, and is suitable for use in psychological research, counseling, educational settings, and the health sciences.

#### 2.4.5 EUROHIS-QOL 8-item index

The EUROHIS-QOL 8-item index is a short form of the WHO Quality of Life scale (WHOQOL), developed by the WHOQOL Schmidt et al. (25) group to assess general quality of life across various life domains. The Turkish adaptation was performed by Eser et al. (26). The scale comprises 8 items addressing physical and psychological health, daily activities, self-esteem, personal relationships, financial resources, living conditions, and overall life satisfaction. Each item is rated on a 5-point Likert scale. Higher scores indicate a better perceived quality of life. The Turkish version demonstrated satisfactory internal consistency (Cronbach’s alpha = 0.78).

#### 2.4.6 Green facility exposure/use (GFE): self-report indicators

Green facility exposure/use was operationalized using a brief set of self-report indicators designed to capture participants’ exposure to and use of nature-based exercise contexts. The indicators were developed based on the conceptual literature on green exercise, nature-based physical activity, and contextual access to green sports environments (10, 11, 14). Rather than functioning as a standalone psychometric scale, the GFE measure was intended to represent a context-specific operational index reflecting participants’ exercise setting, frequency of use, time spent in green environments, and accessibility of nearby green exercise areas.

The content of the indicators was structured to reflect four practical dimensions of green exercise exposure/use: (i) frequency of exercise sessions performed in green are-as, (ii) the most commonly preferred exercise setting, (iii) weekly time spent exercising in green areas, and (iv) access to the nearest green exercise environment. This approach was adopted to ensure that the index captured both behavioral use and environmental access within a single contextual measure.

Before data collection, the indicator set was reviewed for content relevance, conceptual clarity, and contextual appropriateness by field-informed reviewers. In addition, two language experts examined the wording of the items in terms of linguistic clarity and comprehensibility. Minor revisions were made accordingly before the final administration. Because the purpose of this measure was to operationalize contextual green exposure/use rather than to establish a new psychometric scale, the indicators were analyzed as an observed composite index in the structural model.

Indicators (GFE):

1) In the last 7 days, how many of your exercise sessions took place in parks/green areas? (0/1/2/3/4+).
2) Where do you most often exercise? (Indoor facility/Home/Outdoor non-green/Park-green area/Mixed).
3) Approximate weekly time spent exercising in green areas: (0 min/1–59/60–149/150–299/300+).
4) Travel time to the nearest green exercise area: (0–5 min/6–10/11–20/21–30/30+).

Scoring: Responses were coded on a common 0–4 metric, and the item scores were averaged to obtain a GFE index score. An averaged score was preferred over a summed score in order to preserve a common response metric across indicators and to facilitate interpretation, with higher scores indicating greater green facility expo-sure/use.

#### 2.4.7 Smart facility/technology interaction (SFTI): self-report indicators

Smart facility/technology interaction was operationalized using a brief set of self-report indicators designed to capture participants’ engagement with technology-supported exercise tools and digital exercise support features. The indicators were developed based on the conceptual literature on digital exercise monitoring, wearable technology, app-based physical activity tracking, and technology-supported exercise environments. Rather than functioning as a standalone psychometric scale, the SFTI measure was intended to serve as a context-specific operational index of participants’ interactions with smart exercise-related technologies. The SFTI index was newly developed for this study and has not undergone independent psychometric validation prior to inclusion in the structural model. Content validity was examined by field-informed reviewers prior to data collection; however, construct validity (convergent, discriminant) has not been formally established. This is a methodological limitation. Future research should subject the SFTI index to rigorous reliability and validity testing—including CFA on independent samples—before incorporating it in confirmatory structural analyses.

The content of the indicators was structured to reflect four practical dimensions of smart exercise-related interaction: (i) use of wearable tracking devices, (ii) use of mobile exercise applications, (iii) frequency of monitoring exercise-related data, and (iv) encounter with or use of digital exercise support tools in exercise settings. This approach was adopted to ensure that the index captured both personal technology use and environmental interaction within a single contextual measure.

Before data collection, the indicator set was reviewed for content relevance, conceptual clarity, and contextual appropriateness by field-informed reviewers. In addition, two language experts examined the wording of the items for linguistic clarity and comprehensibility. Minor revisions were made accordingly before the final administration. Because the purpose of this measure was to operationalize contextual smart facility/technology interaction rather than to establish a new psychometric scale, the indicators were analyzed as an observed composite index in the structural model.

Indicators (SFTI):

1) Do you use a smartwatch/fitness band during exercise? (Yes/No).
2) Do you use a mobile app to track exercise (e.g., Strava, Nike Run Club, Google Fit)? (Yes/No).
3) How often do you monitor exercise data (steps, heart rate, duration, pace)? (Never/Rarely/Sometimes/Often/Always).
4) How often do you encounter/use digital exercise support tools in the exercise area (e.g., QR-based boards, smart bike systems, app-based guidance where avail-able)? (Never/Rarely/Sometimes/Often/Always).

Scoring: To ensure metric comparability across indicators, binary items were re-scaled to match the 0–4 response metric used for the ordinal items (No = 0, Yes = 4). Frequency-based items were coded from 0 to 4. All indicators were aligned in the same direction and averaged to obtain a composite SFTI index score, with higher scores indicating greater interaction with smart exercise technologies.

### 2.5 Data analysis

The data collected through the online survey were analyzed using IBM SPSS 26.0 and AMOS 26.0. Prior to hypothesis testing, the dataset was examined for missing values, outliers, and multivariate normality assumptions. Missing data were below 2% and handled using full-information maximum likelihood (FIML) in AMOS. Standardized z-scores and Mahalanobis distances were used to detect outliers; no extreme cases were identified. Descriptive statistics (means, standard deviations, frequencies, and percentages) were computed to summarize participant characteristics and study variables. Reliability was assessed using Cronbach’s alpha (*α*) and McDonald’s omega (*ω*), with values ≥0.70 considered acceptable. Construct validity was evaluated using Confirmatory Factor Analysis (CFA) only for the validated psychometric scales (SMS-II, PSS-10, BRS, and EUROHIS-QOL8). Convergent validity for these scales was evaluated using Average Variance Extracted (AVE; >0.50) and Composite Reliability (CR; >0.70). Green exposure/use (GFE) and smart technology interaction (SFTI) were treated as operational indices, computed from coded indicator items (averaged scores), and included as observed variables rather than latent measurement constructs. Because GFE, SFTI, and physical activity were entered as observed/composite variables while the validated psychometric scales (SMS-II, PSS-10, BRS, and EUROHIS-QOL8) were modeled as latent constructs, the analysis constitutes a hybrid structural model that combines latent and observed (path-analytic) components rather than a fully latent-variable SEM. IPAQ-SF responses were converted into total physical activity (MET-min/week) using standard IPAQ scoring procedures and included as an observed variable. Model fit was evaluated using multiple indices (*χ*^2^/d*f*, CFI, TLI, RMSEA, and SRMR), with commonly used cutoffs (*χ*^2^/d*f* < 3, CFI/TLI > 0.90, RMSEA < 0.08, SRMR < 0.08). Pearson correlation analysis was performed to examine bivariate associations among key variables. Finally, SEM was conducted in AMOS using maximum likelihood estimation, and standardized path coefficients were reported. Indirect effects were tested using bootstrap resampling (5,000 samples) with 95% confidence intervals. The assumptions and conditions for SEM were evaluated prior to analysis: (1) Sample adequacy: *N* = 300 exceeds the recommended 5–10 participants per estimated parameter. (2) Multivariate normality: Mardia’s normalized multivariate kurtosis was computed (Mardia’s kurtosis = 4.21, critical ratio = 2.88), indicating acceptable levels for maximum likelihood estimation (16). (3) Pearson correlations were used at the composite-score level; although scale items use ordinal response formats, composite total scores demonstrated approximately continuous, normally distributed distributions, supporting the use of Pearson *r* for bivariate preliminary analyses (27). (4) Model identification was confirmed with positive degrees of freedom ensuring estimability of all free parameters.

## 3 Results

### 3.1 Preliminary analyses and measurement results

The final dataset included *N* = 300 participants. Normality assumptions were met, with all skewness and kurtosis values within the acceptable range of ±2. Multicollinearity was not a concern, as all Variance Inflation Factors (VIFs) were below 5. These findings confirmed that the data set was appropriate for Structural Equation Modeling (SEM).

Table 1 presents the results of reliability and confirmatory factor analysis (CFA) for each scale. Internal consistency was acceptable to excellent, with Cronbach’s *α* ranging from 0.80 to 0.88 and McDonald’s *ω* ranging from 0.81 to 0.89. Construct validity indicators also supported the model, with Average Variance Extracted (AVE) exceeding the 0.50 threshold and Composite Reliability (CR) above 0.70 for all scales. Standardized factor loadings ranged from 0.58 to 0.85, indicating good item convergence. Although Table 1 focuses on scale-level reliability (α, ω) and item-level CFA parameters (loadings, AVE, CR), model fit for each individual scale CFA was as follows: SMS-II: *χ*^2^/d*f* = 2.31, CFI = 0.95, RMSEA = 0.066; PSS-10: *χ*^2^/d*f* = 1.98, CFI = 0.97, RMSEA = 0.057; BRS: *χ*^2^/d*f* = 1.74, CFI = 0.98, RMSEA = 0.049; EUROHIS-QOL8: *χ*^2^/d*f* = 2.12, CFI = 0.96, RMSEA = 0.061. All individual CFAs demonstrated acceptable-to-good fit prior to inclusion in the structural model.

**Table 1:** Reliability and CFA results for the validated psychometric scales.

Scale	*α* (Cronbach)	*ω* (McDonald)	AVE	CR	Factor loadings
SMS-II	0.87	0.89	0.60	0.84	0.65–0.85
PSS-10	0.88	0.89	0.57	0.85	0.61–0.78
BRS	0.80	0.81	0.53	0.76	0.58–0.74
EUROHIS-QOL8	0.85	0.86	0.59	0.83	0.60–0.79

CFA, AVE, and CR values are reported for the validated psychometric scales only.

### 3.2 Descriptive statistics and correlations

Table 2 presents means, standard deviations, and inter-correlations among the study variables, including green facility exposure/use (GFE), smart facility/technology interaction (SFTI), physical activity (IPAQ-SF), sport motivation (SMS-II), perceived stress (PSS-10), psychological resilience (BRS), and quality of life (EUROHIS-QOL8). Correlations were generally in the expected directions and were consistent with the structural model.

**Table 2:** Descriptive statistics and correlations between variables (*N* = 300).

Variable	*M*	SD	1	2	3	4	5	6	7
1. IPAQ (total MET-min/week)	1427.79	1206.82	–						
2. SMS-II	3.04	0.51	0.35^**^	–					
3. PSS-10	2.98	0.43	−0.20^**^	−0.25^**^	–				
4. BRS	3.02	0.31	0.20^**^	0.30^**^	−0.30^**^	–			
5. QOL8	3.02	0.33	0.15^**^	0.25^**^	−0.20^**^	0.45^**^	–		
6. GFE (index mean)	2.07	0.65	0.18^**^	0.20^**^	−0.10^*^	0.12^*^	0.18^**^	–	
7. SFTI (index mean)	1.33	0.51	0.12^*^	0.18^**^	−0.05	−0.08	0.10^*^	0.15^**^	–

*p < 0.05; **p < 0.01. IPAQ values represent total physical activity in MET-min/week (walking + moderate + vigorous). GFE, green facility exposure/use index (mean of coded indicators); SFTI, smart facility/technology interaction index (mean of coded indicators).

### 3.3 Structural model results

The structural model demonstrated acceptable-to-good fit: *χ*^2^/d*f* = 2.10, CFI = 0.96, TLI = 0.95, RMSEA = 0.051, and SRMR = 0.044. Overall, these indices met commonly used cutoff criteria, indicating that the model provided an adequate representation of the observed data (28).

**Figure 1:**
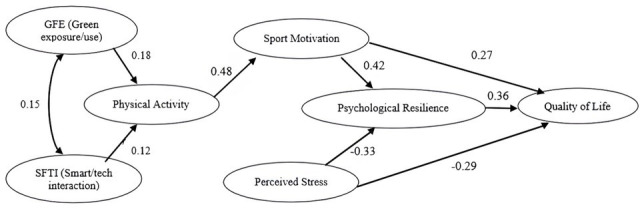
Structural model with standardized path coefficients. All coefficients are standardized. Solid lines represent statistically significant paths at *p* < 0.05. Model fit: *χ*^2^/d*f* = 2.10, CFI = 0.96, TLI = 0.95, RMSEA = 0.051, and SRMR = 0.044.

Figure 1 presents the standardized path coefficients of the SEM model.

The standardized structural path coefficients are shown in Figure 1 and summarized in Table 3. Green facility exposure/use (GFE) and smart facility/technology interaction (SFTI) showed small positive associations with physical activity (IPAQ total MET-min/week): GFE → Physical Activity (*β* = 0.18); SFTI → Physical Activity (*β* = 0.12). Physical activity positively predicted sport motivation (*β* = 0.48), and sport motivation positively predicted psychological resilience (*β* = 0.42). Psychological resilience positively predicted quality of life (*β* = 0.36), and sport motivation also had a direct positive effect on quality of life (*β* = 0.27). Perceived stress negatively predicted psychological resilience (*β* = −0.33) and quality of life (*β* = −0.29). Exogenous predictors were allowed to covary (GFE ↔ SFTI = 0.15). Direct and indirect pathways are summarized in Table 3. Several paths—particularly stress-related paths—did not reach *p* < 0.05 in the ML estimation (shown as dotted lines in Figure 1). These paths were retained on theoretical grounds: the negative role of perceived stress on resilience and quality of life is consistently supported in the stress-and-coping literature (29). Removing theoretically justified non-significant paths would produce a mis-specified model. Non-significance may partly reflect limited power for smaller stress-related effects in the present sample (*N* = 300).

**Table 3:** Structural path coefficients (standardized estimates).

Path	Estimate (*β*)	SE	CR	*p*
GFE → PA	0.18	0.04	4.50	0.001
SFTI → PA	0.12	0.04	3.00	0.003
PA → SM	0.48	0.05	8.60	<0.001
SM → PR	0.42	0.05	6.02	<0.001
PR → QoL	0.36	0.06	6.02	<0.001
SM → QoL	0.27	0.05	10.40	<0.001
PS → PR	−0.33	0.07	−4.14	0.001
PS → QoL	−0.29	0.06	−2.83	0.005

GFE, green facility exposure/use; SFTI, smart facility/technology interaction; PA, physical activity; SM, sport motivation; PR, psychological resilience; QoL, quality of life; SE, standard error; CR, critical ratio.

### 3.4 Indirect effects

To assess indirect effects, a bootstrap analysis with 5,000 samples was conducted. Results indicated that both sport motivation and psychological resilience significantly mediated the relationship between physical activity and quality of life. In addition, green facility exposure/use and smart facility/technology interaction showed significant indirect associations with quality of life through physical activity, sport motivation, and psychological resilience.

For fully mediated pathways, total effects and indirect effects are mathematically equivalent. The total effects column is retained in Table 4 for completeness and to enable comparison with partially mediated pathways in the same table. Readers should note that for fully mediated paths, the indirect effect column alone is sufficient.

**Table 4:** Bootstrap estimates of indirect and total effects among the study variables (*N* = 300).

Pathway	Total effect	Indirect effect	SE	95% CI lower limit	95% CI upper limit
GFE → PA → SM → PR → QoL	0.041	0.041	0.007	0.028	0.056
SFTI → PA → SM → PR → QoL	0.028	0.028	0.009	0.012	0.047
PA → SM → PR → QoL	0.432	0.214	0.025	0.169	0.273
SM → PR → QoL	0.317	0.142	0.021	0.104	0.186
PA → SM → QoL	0.228	0.093	0.019	0.056	0.132

GFE, green facility exposure/use; SFTI, smart facility/technology interaction; PA, physical activity; SM, sport motivation; PR, psychological resilience; QoL, quality of life; SE, standard error; CI, confidence interval. All indirect effects were statistically significant at *p* < 0.01, based on 5,000 bootstrap samples.

## 4 Discussion

This study examined the relationships among physical activity, sport motivation, perceived stress, psychological resilience, and quality of life within a structural model that also incorporated green facility exposure/use and smart facility/technology interaction. Overall, the findings were consistent with the proposed structural model and with relevant theoretical perspectives in sport and health psychology. It is important to emphasize that the cross-sectional design and simultaneous measurement of all variables through self-report preclude causal interpretations. The SEM paths reflect correlational associations rather than directional causal effects. Alternative orderings are equally plausible: motivated individuals may engage in more physical activity; resilient individuals may maintain exercise more readily; and higher quality of life may facilitate participation. All findings should therefore be interpreted as correlational patterns.

One of the main findings of the study was that higher physical activity was associated with higher sport motivation. This pattern is broadly consistent with self-determination theory (30, 31), which suggests that behavior is more likely to be sustained when individuals experience autonomy, competence, and relatedness. In this context, regular physical activity may be associated with stronger motivation to participate in sport. This interpretation is also in line with previous research indicating that physically active individuals tend to report more favorable psychological and behavioral outcomes (4, 32, 33).

The findings further showed that sport motivation was positively associated with psychological resilience and quality of life. This suggests that motivation may play an important explanatory role in understanding how physically active individuals maintain more adaptive psychological functioning. Earlier research has similarly emphasized the importance of motivational processes in supporting resilience and well-being in sport settings (34). In the present study, motivation appeared to function as one of the key pathways linking physical activity to more favorable psychological outcomes. Motivational and other positive psychological factors have also been identified as correlates of sport persistence (39).

Another important finding was that perceived stress was negatively associated with both psychological resilience and quality of life. This result is consistent with the stress and coping framework of Lazarus and Folkman (29) and with previous work showing that stress undermines well-being and adaptive functioning (21, 35). At the same time, the positive association between resilience and quality of life supports the view that resilience may serve as a protective psychological resource under stress and is consistent with meta-analytic evidence linking trait resilience to better mental health (40). Taken together, these findings suggest that individuals reporting lower stress and higher resilience also tend to report better overall quality of life.

A notable contribution of the study is the evidence for indirect pathways. The findings indicated that physical activity was indirectly associated with quality of life through sport motivation and psychological resilience. This stepwise pattern is important because it suggests that the relationship between physical activity and quality of life may be explained not only by behavioral engagement itself, but also by the motivational and adaptive psychological processes accompanying that engagement. This interpretation is consistent with previous studies reporting mediating roles for resilience and related psychological mechanisms in the relationship between physical activity and well-being (7, 36, 37). This interpretation is also supported by recent evidence showing that adults with moderate physical activity levels reported significantly better quality of life outcomes than those with low physical activity levels, further underscoring the importance of active lifestyles for sustainable health promotion (6).

The study also contributes to the growing literature on green exercise and technology supported physical activity by incorporating green facility exposure/use and smart facility/technology interaction into the same structural model. The results showed that both green and smart exercise-related contexts were positively associated with physical activity, although these associations were relatively small. This is still meaningful, as green and smart contexts may operate as contextual facilitators rather than direct determinants of psychological outcomes. In other words, access to nature based exercise settings and interaction with smart exercise technologies may support participation in physical activity, which in turn may be linked to more favor-able outcomes through sport motivation and psychological resilience. In this respect, the study offers a more integrated perspective than approaches that examine green exercise or smart exercise environments separately (10, 11, 14). This interpretation is further supported by recent evidence showing that a sustainable training model combining supervised outdoor exercise with telecoaching can produce beneficial outcomes, highlighting the potential value of integrating nature-based and technology-supported exercise approaches within sustainable health promotion (38).

From a sustainability perspective, these findings are relevant for the design of exercise environments and public health strategies. Sustainable sport and recreation planning should not focus only on the availability of exercise spaces, but also on how these spaces encourage long-term participation, accessibility, and supportive user experience. Nature-based exercise opportunities and technology-supported features may together contribute to more sustainable active-living environments by supporting regular participation in physical activity and, indirectly, psychological well-being.

### 4.1 Limitations and implications

This study has several limitations. First, the cross-sectional design does not allow causal inferences. Second, all variables were measured using self-report instruments, which may be subject to recall and social desirability biases. Third, the use of convenience sampling limits the generalizability of the findings. Fourth, the GFE and SFTI measures were operational indices developed for this study rather than fully validated psychometric scales. Future research should test the proposed relationships using longitudinal or experimental designs, more diverse samples, and more robust and con-text-sensitive environmental measures. Despite these limitations, the findings offer useful implications for urban recreation planning, public health policy, and the development of sustainable sport environments that integrate both nature-based and technology-supported exercise opportunities.

An additional sampling limitation warrants emphasis. Online recruitment via social media and sport-related networks likely over-represents digitally active, health-conscious individuals already engaged in physical activity. Given that smart facility/technology interaction (SFTI) was a central construct, this selection bias is particularly consequential: individuals with lower digital engagement or limited access to green spaces may be systematically underrepresented, constraining the generalizability of findings to broader and more diverse populations.

## 5 Conclusion

This study showed that physical activity was positively associated with sport motivation, which in turn was associated with psychological resilience and quality of life. Perceived stress was negatively associated with both resilience and quality of life, whereas resilience was positively associated with quality of life. Green facility expo-sure/use and smart facility/technology interaction were also positively associated with physical activity and showed indirect links with quality of life through motivational and psychological pathways.

Overall, the findings suggest that sustainable sport and exercise environments should be considered not only in physical or infrastructural terms, but also in relation to the motivational and psychological processes they may support. Nature-based and technology-supported exercise opportunities may promote more sustainable active lifestyles and indirectly contribute to improved well-being and quality of life.

## Data Availability

The raw data supporting the conclusions of this article will be made available by the authors, without undue reservation.
